# Decoding manipulative narratives in cognitive warfare: a case study of the Russia-Ukraine conflict

**DOI:** 10.3389/frai.2025.1566022

**Published:** 2025-09-11

**Authors:** Andrii Paziuk, Dmytro Lande, Elina Shnurko-Tabakova, Phillip Kingston

**Affiliations:** ^1^Law and International Relations Faculty, State University Kyiv Aviation Institute, Kyiv, Ukraine; ^2^Department of Information Security, National Technical University of Ukraine “Igor Sikorsky Kyiv Polytechnic Institute,” Kyiv, Ukraine; ^3^International Institute of Cyber Diplomacy and AI Security, Law and International Relations Faculty, State University Kyiv Aviation Institute, Kyiv, Ukraine

**Keywords:** cognitive warfare, semantic analysis, Russia-Ukraine conflict, nuclear threat narratives, emotional manipulation, disinformation

## Abstract

**Introduction:**

This study investigates the construction and dissemination of manipulative narratives in the context of cognitive warfare during the Russia-Ukraine conflict. Leveraging a mixed-methods approach that integrates AI-assisted semantic analysis with expert validation, we examine how adversarial messaging exploits cognitive biases-such as fear and confirmation bias-to influence perceptions and disrupt institutional trust.

**Methods:**

Using the proprietary Attack-Index tool and large language models (LLMs), we detect linguistic markers of manipulation, including euphemisms, sarcasm, and strategic framing.

**Results:**

Our findings demonstrate that emotionally charged narratives, particularly those invoking nuclear threat scenarios, are synchronized with key geopolitical events to influence decision-makers and public opinion. The study identifies five thematic clusters and traces shifts in rhetorical strategies over time, showing how manipulative discourse adapts to geopolitical contexts. Special attention is given to the differentiated targeting of international political elites, Western publics, and Russian domestic audiences, each exhibiting varied cognitive vulnerabilities.

**Discussion:**

We acknowledge methodological and ethical limitations, including the dual-use potential of AI tools and challenges in establishing causal inferences. Nonetheless, this study offers the following key contributions:

1. Empirically establishing nuclear rhetoric as a strategic element of narrative manipulation, particularly around NATO summits and military aid announcements.
2. Advancing an integrated analytical framework that combines semantic clustering and AI-based discourse detection to monitor information threats in real time.
3. Providing actionable insights for policy and digital security, including the development of countermeasures and international collaboration in addressing cognitive warfare.

## 1 Introduction

Cognitive warfare represents a new frontier in the evolution of conflict, where the mind itself becomes the primary battleground. Unlike traditional kinetic warfare, cognitive warfare operates in the psychological and informational domains, exploiting vulnerabilities in human cognition to manipulate beliefs, emotions, and decision-making processes. NATO describes it as a deliberate effort to influence, disrupt, or protect the cognitive processes of individuals and societies, highlighting its growing role in modern hybrid threats (Deppe and Schaal, 2024). This emergent form of warfare transcends conventional propaganda and psychological operations by incorporating advanced technologies and strategic timing to maximize its impact. Cognitive warfare has been recognized as a critical element of hybrid threats, which combine conventional, unconventional, and technological methods to achieve strategic objectives. Its operational methods include the dissemination of disinformation, narrative framing, emotional manipulation, and exploiting cognitive biases such as confirmation bias and anchoring. The strategic use of these techniques is evident in modern conflicts, where adversaries target individuals and societal structures to destabilize trust in institutions and polarize populations. NATO's reports emphasize the role of cognitive warfare in shaping perceptions during geopolitical conflicts, highlighting its integration with cyber operations and kinetic attacks (NATO Allied Command Transformation, 2023). This multifaceted approach leverages digital ecosystems to create information asymmetry, where adversaries overwhelm their targets with a flood of narratives, some true, some false, and some a blend of both. By doing so, they obscure the truth, manipulate public opinion, and erode trust in democratic institutions. This tactic has been prominently observed in the Russia-Ukraine conflict, where disinformation campaigns have been used to undermine support for Ukraine and amplify divisions within NATO and the European Union (Digital Forensic Research Lab, 2023). Technological advancements, particularly in artificial intelligence, big data analytics, and social media algorithms, have amplified the reach and precision of cognitive warfare. Platforms like X (formerly Twitter) and Facebook have become battlegrounds for influencing public discourse, with adversaries employing bot networks, troll farms, and deepfake technologies to propagate narratives. These operations are reactive and proactive, often synchronizing their efforts with significant geopolitical events such as elections, military exercises, or international summits (Marsili, 2023). The ability to control the narrative in these moments can significantly influence electoral outcomes, consultative democratic processes, direct democracy, policy decisions and support, alliances, traditional media, and overall public support.

### 1.1 Significance of the research

The digital age has exponentially increased the scope and impact of cognitive warfare, making it a critical area of study for ensuring geopolitical stability. As societies become more interconnected through digital platforms, the opportunities for adversaries to exploit cognitive vulnerabilities have grown. Cognitive warfare poses unique challenges because its effects are not always immediately visible. Unlike physical attacks, the damage inflicted by cognitive operations is often psychological and societal, manifesting over time as declining trust, increasing polarization, and eroding democratic norms. The importance of understanding cognitive warfare lies in its capacity to disrupt the foundational elements of liberal democracies. Cognitive warfare undermines the social contract that binds societies together by targeting trust. In a world where information is currency, the ability to control or manipulate narratives becomes a powerful weapon. This has been demonstrated in conflicts such as the Russia-Ukraine war, where disinformation campaigns have aimed to delegitimize Ukrainian leadership, weaken international support for Ukraine, and sow discord among NATO allies. Examples such as claims of Ukraine planning to use a “dirty bomb” to frame Russia illustrate the tactical deployment of fear-inducing narratives designed to polarize international opinion. Moreover, cognitive warfare extends beyond the battlefield, affecting policymaking, election outcomes, and international relations. Governments and organizations must recognize that cognitive warfare is not just a military issue but a societal one, requiring a whole-of-society response. Media literacy programs, public awareness campaigns, and collaboration between governments, academia, and the private sector are essential to building resilience against cognitive attacks. This research significantly advances the understanding of cognitive warfare, particularly its application during the Russia-Ukraine conflict. It identifies nuclear rhetoric as a pivotal strategic tool within manipulative narratives, demonstrating their synchronization with geopolitical milestones such as NATO summits and military aid announcements. By introducing an innovative analytical framework, which combines semantic analysis and AI-driven methodologies, the study enhances the detection and prediction of disinformation narratives, providing actionable insights for countering their psychological and geopolitical impacts. Central to this research is the concept of leveraging predictive analytics in cognitive warfare. Tools like the Attack Index and advanced AI-driven methodologies are employed to forecast manipulative narrative trends, bridging the gap between retrospective analysis and proactive strategic planning. This approach not only reveals the mechanisms of cognitive operations but also underscores the necessity of aligning counter-narratives and policy responses preemptively. Through the lens of the Russia-Ukraine conflict, the study illustrates how cognitive warfare strategically employs fear and uncertainty, particularly through nuclear rhetoric, to manipulate public discourse and achieve geopolitical objectives. This synchronization of manipulative narratives with critical geopolitical events exemplifies the precision and adaptability of modern cognitive warfare tactics.

### 1.2 Research gap

While the study of cognitive warfare has gained traction in recent years, significant gaps remain in the literature. Much of the existing research focuses on the tactical aspects of disinformation, such as the dissemination methods and the role of social media platforms. However, there is a need for a more comprehensive understanding of the strategic dimensions of cognitive warfare, particularly its integration with other elements of hybrid threats, such as cyber operations and economic coercion. One underexplored area is the synchronization of cognitive operations with geopolitical milestones. For example, during the Russia-Ukraine conflict, disinformation campaigns have been carefully timed to coincide with NATO summits, military aid announcements, and key elections in Western democracies. This strategic timing magnifies the impact of cognitive operations by aligning them with moments of heightened public and political attention. Understanding how adversaries coordinate these efforts is crucial for developing effective countermeasures (Marsili, 2023).

Another gap lies in the study of emotional manipulation in cognitive warfare. While much attention has been given to the content of disinformation, less has been said about its emotional appeal. Adversaries often craft narratives that evoke fear, anger, or resentment, knowing that emotions significantly shape beliefs and behaviors. For instance, during the Russia-Ukraine war, disinformation campaigns have exploited fears of nuclear escalation and economic instability to influence public opinion in Europe and the United States (Splidsboel Hansen, 2021). Researching the emotional dimensions of cognitive warfare can provide deeper insights into its effectiveness and inform the development of more nuanced counter-narratives. Finally, there is a need to explore the role of emerging technologies in detecting and countering cognitive warfare. Semantic analysis, sentiment detection, and narrative mapping have shown promise in identifying disinformation patterns. However, these tools must be integrated into a broader framework that accounts for cognitive operations' strategic and emotional dimensions. Developing such a framework is essential for staying ahead in this evolving domain of conflict.

### 1.3 Research objectives

This research aims to address these gaps by examining the mechanisms, impacts, and countermeasures of cognitive warfare, focusing on the Russia-Ukraine conflict. The specific objectives are as follows:

1. Analyze the mechanisms of cognitive warfare: investigate how adversaries use narrative framing, emotional manipulation, and cognitive biases to influence public opinion and decision-making.
2. Explore the strategic integration of cognitive operations: examine how cognitive warfare is synchronized with other elements of hybrid threats, such as cyberattacks and economic coercion, to maximize its impact.
3. Evaluate the role of timing and context: study the timing of cognitive operations about geopolitical events, identifying patterns and strategies used by adversaries.
4. Propose countermeasures and resilience-building strategies: develop recommendations for governments, organizations, and societies to enhance their resilience against cognitive warfare, focusing on public awareness, media literacy, and advanced analytical tools.

## 2 Literature review

### 2.1 Foundational theories of cognitive warfare

Cognitive warfare represents a pivotal evolution in strategic conflict, blurring the lines between psychological operations, information warfare, and hybrid threats. Rooted in psychological theories of influence and propaganda, cognitive warfare leverages advances in digital technology to target the cognitive vulnerabilities of individuals and societies (Bilal, 2021). This framework has been extensively discussed by Deppe and Schaal (2024), who describe cognitive warfare as an “existential threat to the cognitive domain,” where perception becomes the primary battleground. Building on foundational theories, NATO's reports outline how cognitive warfare integrates with hybrid strategies, targeting systemic and individual-level cognition to create asymmetries in trust, perception, and decision-making (NATO Allied Command Transformation, 2024). The hybrid warfare lens, as outlined by Weissmann et al. (2021), situates cognitive warfare within a broader spectrum of unconventional conflict, merging cyber, kinetic, and cognitive strategies. Historical antecedents of cognitive warfare can be traced to Cold War psychological operations, where state actors employed propaganda to influence ideological allegiance. Buzan and Waever (2003) theories on securitization add context to the weaponization of narratives, framing them as tools to mobilize collective fears and justify political actions. These theoretical underpinnings have gained renewed relevance in the digital era, where social media and artificial intelligence amplify the reach and effectiveness of cognitive warfare (Ngwainmbi, 2024).

### 2.2 Mechanisms of cognitive warfare

#### 2.2.1 Psychological manipulation

Psychological manipulation remains a cornerstone of cognitive warfare, targeting emotional and cognitive vulnerabilities to induce behavioral and perceptual shifts. Fear, anger, and existential threats are central to this strategy. Henschke (2025) argues that psychological manipulation in cognitive warfare often operates at a subconscious level, leveraging emotional salience to bypass rational scrutiny. In the context of the Russia-Ukraine conflict, psychological manipulation has been used to frame existential threats, such as NATO's alleged encroachment on Russian sovereignty, as a means to justify aggressive actions. Gambhir (2016) underscores the parallels between these tactics and ISIS's use of fear-based narratives, which targeted vulnerabilities in Western societies to radicalize individuals and undermine public confidence.

#### 2.2.2 Narrative synchronization

Narrative synchronization refers to aligning manipulative narratives with key geopolitical events, enhancing their resonance and perceived legitimacy. Jensen and Ramjee (2023) highlight how Russian narratives during the Ukraine crisis synchronized with NATO summits and Western military aid announcements, framing these actions as provocations. This synchronization creates temporal relevance, which amplifies the perceived credibility of disinformation. Marsili (2023) describes how narrative synchronization is facilitated through algorithmic targeting, ensuring that key messages reach specific audiences at opportune moments. By aligning narratives with tangible events, such as energy shortages or sanctions, adversaries create a feedback loop that reinforces public skepticism and divisions.

#### 2.2.3 Exploitation of cognitive biases

Exploiting cognitive biases such as confirmation bias, anchoring, and the availability heuristic is integral to cognitive warfare strategies. Nguyen (2023) explains that these biases predispose individuals to accept disinformation that aligns with their pre-existing beliefs, making them more susceptible to manipulation. Extensive research, including Benkler et al. (2018) and Bradshaw and Howard (2019), demonstrates how state-sponsored disinformation exploits cognitive biases to polarize audiences and erode trust in democratic institutions. Plaza et al. (2023) identify emotional contagion as a related mechanism, where emotionally charged narratives spread rapidly through social networks, amplifying their impact. This dynamic highlights the interplay between psychological vulnerabilities and digital platforms in cognitive warfare. Lewandowsky et al. (2017) highlight mechanisms that make disinformation effective and argue that simple, emotionally resonant narratives are more easily processed and remembered by individuals than complex, nuanced truths. This cognitive preference for simplicity makes populations more susceptible to accepting and spreading disinformation, especially when the information is intricate and demands more significant cognitive effort to understand.

### 2.3 Role of AI in amplifying cognitive warfare

#### 2.3.1 AI-driven content creation

Artificial intelligence has revolutionized cognitive warfare by enabling the creation of highly convincing and scalable manipulative content. Deepfakes, synthetic media, and automated narrative generation tools allow adversaries to craft persuasive disinformation with minimal effort (Henschke, 2025). For example, AI-generated deepfake videos of Ukrainian leaders were used to undermine public confidence and create confusion during the early stages of the conflict (Fisher et al., 2024).

#### 2.3.2 Sentiment analysis and targeted messaging

AI tools like sentiment analysis play a dual role in cognitive warfare. On one hand, they enable adversaries to monitor public sentiment and tailor narratives to resonate with emotional undercurrents. Huang et al. (2024) describe how sentiment analysis algorithms were used to detect and exploit shifts in public opinion during geopolitical crises. On the other hand, these tools empower counter-cognitive operations, enabling real-time detection of disinformation trends (Index Systems Ltd., 2023). Modern AI has enabled a deeper understanding of the propagation of messaging through the social graph that underpins social networks. This enriches the ability to granularly segment users and their patterns of influence, and personalize disinformation to that segment and its most effective delivery mechanism.

#### 2.3.3 Strategic amplification of narratives

The scalability of AI-driven tools amplifies the reach and impact of disinformation campaigns. Nimmo and Flossman (2024) highlights the role of large-scale language models in generating tailored narratives that exploit cultural and ideological divisions. This strategic deployment creates a multiplier effect, where the same narrative resonates across diverse audiences, maximizing its disruptive potential.

### 2.4 Countermeasures against cognitive warfare

#### 2.4.1 Technological interventions

Technological solutions form the backbone of counter-cognitive warfare strategies. Attack-Index allows real-time monitoring of adversarial narratives, enabling rapid response to disinformation campaigns. Additionally, AI-driven detection systems identify linguistic patterns and anomalies that indicate manipulative intent (Huang et al., 2024).

#### 2.4.2 Media literacy and public awareness

Building societal resilience through media literacy programs is essential to countering cognitive warfare. NATO Allied Command Transformation (2023) emphasizes the role of public education initiatives in equipping individuals with the critical thinking skills needed to identify and resist disinformation. Sarwono (2022) underscores the importance of targeting these programs at vulnerable demographics, such as youth and digitally marginalized populations, who are disproportionately affected by manipulative narratives.

#### 2.4.3 Policy and collaborative frameworks

International collaboration is critical in addressing the transnational nature of cognitive warfare. Orinx and Struye de Swielande (2022) call for harmonized policy frameworks that standardize responses to cognitive threats across allied nations. These frameworks enhance collective resilience and serve as deterrents by increasing the costs of engaging in cognitive warfare. The literature on cognitive warfare reveals its central role in shaping modern geopolitical conflicts, emphasizing its evolution from traditional propaganda to highly sophisticated digital strategies. Foundational works such as NATO's Cognitive Warfare Framework and other key contributions (e.g., NATO Allied Command Transformation, 2024; Splidsboel Hansen, 2021) contextualize cognitive warfare as a method for undermining trust, polarizing societies, and destabilizing political structures. Key mechanisms of cognitive warfare—psychological manipulation, narrative synchronization, and emotional exploitation—are strategically employed to amplify societal vulnerabilities. Studies such as Huang et al. (2024) and Grindrod (2024) demonstrate how emotional exploitation targets individual biases, including confirmation bias and availability heuristics, leveraging these tendencies to promote divisive narratives. By aligning narratives with pivotal geopolitical events, adversaries create a feedback loop that strengthens disinformation and delays collective responses (Brusylovska and Maksymenko, 2023). The integration of advanced technologies further elevates the efficacy of cognitive warfare. Works by Nguyen (2023) and Nimmo and Flossman (2024) illustrate how tools like deepfakes, sentiment analysis, and algorithm-driven narratives enhance the precision of disinformation campaigns. These tools are instrumental in geopolitical conflicts, such as the Russia-Ukraine war, where adversaries use AI-driven methods to erode public trust and manipulate global perceptions (Benkő and Biczók, 2024).

Current countermeasures reflect the complexity of combating cognitive warfare. Media literacy programs (e.g., Wallenius, 2023), real-time monitoring tools like Attack-Index (Index Systems Ltd., 2022, 2023), and AI-driven detection systems are the solutions proposed to mitigate these threats. However, as the review highlights, the adaptability of adversaries and the limitations of current frameworks necessitate ongoing innovation and collaboration across sectors (Jensen and Ramjee, 2023). Benkler et al. (2018) analyze the structure and dynamics of media ecosystems and argue that the commitment to free speech, especially in digital and social media platforms, can inadvertently provide a fertile ground for disinformation campaigns. Furthermore, Bradshaw and Howard (2019) demonstrate how disinformation campaigns evade regulatory attention and enforcement action by masquerading as legitimate political discourse. However, attempts by governments or social media platforms to intervene can paradoxically validate these falsehoods, as regulatory measures are sometimes perceived as acknowledgments of the underlying credibility of the suppressed narratives (DiResta, 2020; Pennycook et al., 2020). Such interventions may reinforce conspiratorial beliefs, leading to increased trust in disinformation among specific populations (Tucker et al., 2017; Marwick and Lewis, 2017). In sum, the literature illustrates the multidimensional nature of cognitive warfare, which intersects psychology, technology, and geopolitics. While advancements in AI and digital platforms have enhanced the reach and impact of manipulative campaigns, they also provide opportunities for countering these threats through detection and resilience-building measures. Future research must focus on bridging existing gaps, particularly integrating interdisciplinary approaches to address the evolving tactics of cognitive warfare. This review underscores the need for a proactive and collaborative global response to safeguard societal stability in the face of this emerging domain.

## 3 Methodology

### 3.1 Research design

This study employs a comprehensive mixed-method approach integrating qualitative and quantitative methodologies to analyze manipulative narratives in cognitive warfare. By combining semantic analysis through the Attack-Index tool with AI-driven narrative analysis, the methodology captures the intricacies of narrative construction, emotional manipulation, and the psychological impact on target audiences. This design bridges theoretical gaps in understanding cognitive warfare and its practical manifestations, providing a holistic lens for examining the intersection of manipulative strategies and technology (Plaza et al., 2023; Deppe and Schaal, 2024; Henschke, 2025).

### 3.2 Rationale for a mixed-method approach

Cognitive warfare involves the interplay of qualitative dimensions—like understanding emotional and rhetorical subtleties—and quantitative dimensions—such as the prevalence, intensity, and temporal dynamics of narratives. The mixed-method approach ensures both depth and breadth, offering nuanced insights into how manipulative narratives evolve and resonate with target audiences (NATO Allied Command Transformation, 2023; Weissmann et al., 2021).

### 3.3 Key components of the research design

#### 3.3.1 Semantic analysis through the attack-index tool

The Attack-Index tool serves as a comprehensive framework for analyzing manipulative narratives by identifying recurring themes, emotional triggers, and narrative trajectories. Its quantitative capabilities measure the frequency and intensity of specific themes, such as nuclear threats, across extensive datasets, offering precise numerical insights into the scale and prevalence of disinformation. Simultaneously, its qualitative features delve into linguistic framing and emotional undertones, revealing the psychological tactics employed in narrative construction (Index Systems Ltd., 2023). The Attack-Index tool was pivotal in uncovering key phraseological patterns and narrative shifts, such as the recurring theme of nuclear aggression, quantified by the tool's clustering algorithm (Appendix). This capability enables the identification of key narrative lines and their dynamics, offering a deeper understanding of how manipulative narratives evolve over time. By clustering related linguistic elements, the tool highlights the structural coherence of disinformation campaigns, aiding in pinpointing central themes and their trajectories (Lande and Shnurko-Tabakova, 2019). As an essential instrument in countering cognitive warfare, the Attack-Index enables the prompt identification of disinformation campaigns, thereby supporting the development of targeted countermeasures. Through its capacity for real-time monitoring and retrospective analysis, it not only helps detect emerging threats but also informs strategic decision-making. The integration of the Attack-Index into broader cognitive warfare frameworks strengthens efforts to neutralize manipulative narratives, safeguard the information environment, and build resilience against information threats (Lande, 2024).

### 3.4 AI-driven narrative analysis

Advanced AI tools, including pre-trained models and Natural Language Processing (NLP) algorithms, supplement semantic analysis by identifying subtler manipulations such as sarcasm, euphemisms, and framing techniques. These tools enhance the detection of cognitive biases and rhetorical tactics embedded in disinformation (Huang et al., 2024; Grindrod, 2024).

### 3.5 Temporal and event-based analysis

By correlating shifts in narratives with geopolitical events, such as NATO summits or military aid announcements, this component provides insights into how manipulative narratives are synchronized with broader strategies to maximize psychological impact (Monaghan, 2020; Benkő and Biczók, 2024).

### 3.6 Data collection: a comprehensive framework

Effective data collection is central to analyzing manipulative narratives in cognitive warfare. This study adopts a systematic, multi-source data collection strategy to ensure the scope and depth necessary for meaningful analysis.

### 3.7 Data sources

The system analyzed around 4,000 Russian websites and 3,000 Telegram channels from November 1, 2022, to March 5, 2023. Using linguistic analysis and machine learning, 36,821 phrases were identified, of which 170 were selected for further analysis. Of these, 170 key phrases—such as “nuclear attack,” “dirty bomb,” “Kyiv authorities,” and “nuclear treaties”—were selected for further analysis. These persistent phrases formed the foundation for constructing a narrative network and uncovering the structural dynamics of disinformation campaigns. Disinformation narratives were categorized into five key clusters, including “Kyiv authorities and the dirty bomb” and “Special operation and nuclear energy,” as visualized in the cluster network map in Appendix Figure 5. This clustering highlights the diversity and strategic focus of Russian narratives.

### 3.8 Preprocessing techniques

Tokenization: text data were parsed into tokens to facilitate semantic and linguistic analysis. Noise filtering: irrelevant or redundant content was removed to ensure analytical rigor. Semantic tagging: data were annotated to categorize emotional tones, themes, and rhetorical devices. Temporal segmentation: data were segmented to analyze narrative evolution over time and correlate shifts with geopolitical events.

### 3.9 Definition of tokens

Let the set of tokens in the network be defined as:


$$T=\left\{t_{1},t_{2},\ldots ,t_{n}\right\}$$

where *t*_*i*_ represents either an individual word in its normalized form (e.g., lemmatized word) or a stable phrase (multi-word expression).

### 3.10 Network representation

The network of phrase interconnections is represented as a graph:


G=(V,E)

where:

- *V* is the set of vertices, corresponding to tokens *T*.
- *E* ⊆ *V* × *V* is the set of edges, representing relationships or co-occurrences between tokens.

### 3.11 Edge weights

Each edge *e*_*ij*_ ∈ *E* has a weight *w*_*ij*_, representing the strength of the connection between tokens *t*_*i*_ and *t*_*j*_. The weight can be calculated based on metrics such as frequency of co-occurrence in the text corpus:


$$w_{ij}=f\left(t_{i},t_{j}\right)$$

where *f*(*t*_*i*_, *t*_*j*_) is the frequency of co-occurrence of *t*_*i*_ and *t*_*j*_ within a defined window size or context.

### 3.12 Cluster analysis

The Gephi environment was employed to analyze narrative data by clustering tokens into modularity classes, thereby identifying distinct themes or “concept classes” within the dataset. The use of network visualizations, such as those generated with Gephi, provided critical insights into narrative interconnections and thematic clustering, revealing how narratives evolved in response to geopolitical developments (Appendix Figure 5). This approach is grounded in graph theory and modularity optimization, enabling the detection of cohesive narrative clusters that reveal the structural dynamics of disinformation campaigns (Bruns and Snee, 2022). Modularity optimization is central to cluster detection. It quantifies the strength of division of a network into clusters (or communities). In this study, various modularity methods were considered (Traag et al., 2019), and the Potts model (Wu, 1982) was specifically applied. The Potts model incorporates a resolution parameter γ, which adjusts the granularity of clustering, allowing the system to determine the optimal number of clusters.

Quality function *H*(*G, P*), or briefly *H*(*P*), for the partition *P* into modules (clusters) of the graph *G* is written as:


$$H\left(P\right)=-\underset{C\in P}{\sum }e_{C}-\gamma \cdot n_{C}^{2},$$

where each cluster *C*∈*P* consists of *e*_*C*_ edges and *n*_*C*_ nodes, and γ is the resolution parameter, which significantly influences the partitioning of the graph into clusters.

### 3.13 Clustering algorithm

The clustering procedure employed within the Gephi environment followed a structured algorithm designed to identify and classify thematic concentrations within the dataset. The integration of modularity clustering and token detection, as detailed in the Appendix, provided a comprehensive framework for analyzing narrative cohesion and thematic evolution across disinformation campaigns. This approach ensured methodological rigor and reproducibility in analyzing the Russian disinformation narratives.

#### 3.13.1 Initialization

Nodes, representing individual tokens or phrases, were preliminarily distributed into clusters based on their initial associations. This step established a foundational configuration for subsequent modularity optimization.

#### 3.13.2 Evaluation

The modularity of the current cluster configuration, denoted as H(P), was calculated. Modularity quantifies the quality of the network partitioning by measuring the strength of connections within clusters relative to those between clusters.

#### 3.13.3 Merging clusters

Nodes or groups of nodes were iteratively merged to enhance modularity. This step optimized the grouping of nodes by forming clusters with strong internal connections.

#### 3.13.4 Iteration

The iterative process of merging and evaluation continued until maximum modularity was achieved, indicating the optimal partitioning of the network. Once modularity ceased to improve, the process was finalized.

#### 3.13.5 Finalization

The algorithm produced well-defined modularity classes, or clusters, characterized by high internal cohesion and minimal external overlap. These clusters encapsulated distinct thematic narratives within the dataset.

### 3.14 Semantic analysis using attack-index

The Attack-Index tool is central to this study's methodology, offering a sophisticated approach to uncovering emotional triggers, key themes, and narrative shifts over time. Designed to track linguistic patterns and emotional cues, the tool provides insights into constructing and disseminating manipulative narratives. Its utility in identifying subtle yet impactful rhetorical devices—such as geopolitical framing and existential threats—is particularly relevant in cognitive warfare. The semantic algorithms employed by the Attack-Index are tailored to detect nuanced narrative shifts, particularly during critical geopolitical events. The procedure involved the following steps:

#### 3.14.1 Identifying recurring motifs

The tool flagged recurring themes such as existential risks, nuclear threats and perceived aggression from Western alliances. These motifs were prevalent in Russian state-controlled media, aligning with broader strategies to provoke fear and justify aggressive policies.

#### 3.14.2 Tracking emotional tone over time

Changes in emotional tone were linked to specific geopolitical events, such as NATO summits or military mobilizations.

#### 3.14.3 Linking themes to events

Semantic tagging connected themes to specific events, such as Western sanctions or high-profile diplomatic meetings. These connections revealed how disinformation was synchronized with real-world developments to amplify its psychological impact.

### 3.15 AI-driven analysis

To complement the insights from the Attack-Index, the study employed AI-driven tools, including pre-trained Large Language Models (LLMs), to analyze the subtleties of manipulative narratives. Unlike traditional semantic tools, LLMs excel at identifying contextual manipulations, such as sarcasm, euphemisms, and logical inconsistencies. These capabilities are crucial for understanding how disinformation exploits psychological vulnerabilities, including biases like confirmation bias and availability heuristics (Grindrod, 2024; Huang et al., 2024).

### 3.16 Detecting linguistic patterns

At the core of the analysis was the deployment of pre-trained AI models designed to detect subtle manipulations embedded within language. These models identify framing techniques that are not immediately apparent to human analysis. For instance, narratives were often framed to downplay acts of aggression while simultaneously amplifying victimhood to evoke empathy or deflect criticism. Such tactics were particularly evident in geopolitical contexts where aggressor nations sought to portray themselves as defenders of sovereignty or morality (Muñoz and Nuño, 2024). By systematically analyzing these patterns, AI tools provided insights into how language can be strategically deployed to manipulate public perception and align with broader disinformation campaigns.

### 3.17 Cross-referencing with semantic analysis

To ensure the robustness and reliability of findings, results derived from AI-driven analysis were cross-referenced with data obtained through semantic tools like the Attack-Index. This validation step was critical for enhancing the credibility of the conclusions drawn from the study. By aligning the thematic and emotional cues identified by AI models with the broader narrative patterns detected through semantic analysis, researchers achieved a cohesive understanding of how disinformation narratives are structured and disseminated. This cross-referencing methodology also minimized the risk of interpretive errors, ensuring that multiple analytical frameworks supported conclusions.

### 3.18 Unveiling biases

A significant component of the procedure involved the examination of biases embedded within disinformation narratives. AI algorithms were employed to uncover cognitive biases, such as confirmation bias or framing effects, which play a pivotal role in influencing target audiences' emotional and psychological responses. These biases were not only instrumental in shaping the narratives but also in enhancing their resonance and persuasiveness. For example, narratives framing international coalitions like NATO as aggressors capitalized on skepticism or distrust among specific demographic groups (Henschke, 2025). By unveiling these biases, the study provided a deeper understanding of the psychological underpinnings that make disinformation campaigns effective. Through these procedural steps, the study explored how manipulative narratives are constructed and perpetuated in cognitive warfare. Integrating AI tools with semantic methodologies provided a robust framework for identifying and analyzing the subtle yet impactful tactics used in modern disinformation efforts. This approach advanced the academic understanding of cognitive warfare and offered actionable insights for countering its psychological and geopolitical impacts.

### 3.19 Triangulation for robustness and reliability

Ensuring the validity and reliability of findings is paramount in any research endeavor, especially when analyzing complex phenomena such as manipulative narratives in cognitive warfare. This study employed triangulation by cross-verifying results derived from the Attack-Index and AI-driven analyses with expert reviews and independent datasets. This method not only bolstered the credibility of the findings but also mitigated potential biases stemming from the analytical tools themselves (Abu Arqoub, 2023; Sarwono, 2022). Experts in cognitive warfare and disinformation provided critical evaluations, helping to refine interpretations and ensure that conclusions aligned with real-world dynamics. Additionally, incorporating independent datasets further strengthened the study's foundation, allowing for comparative analysis and reducing the risk of over-reliance on any single data source.

## 4 Addressing ethical concerns

The ethical dimension of this research was a critical consideration, given the potential sensitivity and impact of findings related to disinformation and cognitive warfare. The study addressed several key ethical challenges:

### 4.1 Data privacy

Protecting individual privacy was a central focus, particularly when handling sensitive datasets from social media and other public platforms. To ensure compliance with ethical standards, anonymized data processing methods were employed. This approach safeguarded the identities of individuals while maintaining the integrity and relevance of the data for analysis (Huntsman et al., 2024).

### 4.2 Bias mitigation in AI tools

AI tools, while powerful, are inherently susceptible to biases that can skew analytical outcomes. To address this, findings from AI analyses were rigorously cross-referenced with expert reviews. This dual-layer validation minimized the influence of AI-induced biases and enhanced the reliability of the results (Nimmo and Flossman, 2024).

## 5 Results and analysis

This section presents the outcomes derived from utilizing the Attack-Index tool and AI-driven language models (LLMs). The focus is on identifying patterns, narratives, and emotional triggers inherent in cognitive warfare, particularly within the context of the Russia-Ukraine conflict.

The research on the Russian Federation's information space during two distinct periods—November 1, 2022 to March 5, 2023, and January to December 2024 utilized data from 4,000 Russian websites and 3,000 Telegram channels. This analysis revealed that nuclear threats remain a consistent and prominent theme within the Russian Federation's information landscape. It is deeply embedded in the primary propaganda narratives of the Russian Federation and reflected in the international segment of the Attack Index service's database.

**Figure 1:**
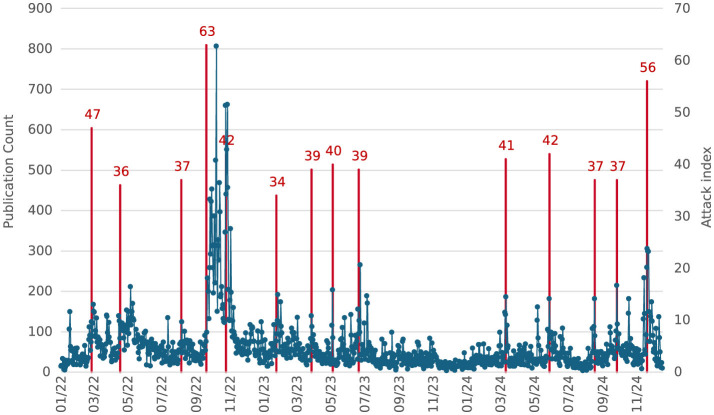
Information dynamics of mentions of possible nuclear strikes in Ukraine.

Figure 1 highlights the dynamic nature of the nuclear threat mentioned in Russian propaganda. The graph captures the daily volume of publications on this topic, showing significant fluctuations and resonant peaks. Red lines mark instances where the Attack Index exceeded 30, signifying periods of heightened propaganda activity. The data indicates that October 2022 experienced the highest peak in information dynamics, with 807 daily mentions, representing the absolute maximum within the analyzed timeframe.

### 5.1 Attack-index findings

Using the clustering methodology outlined in the Appendix, five primary clusters were identified, each representing a significant thematic component of Russian disinformation narratives. These clusters highlight the strategic deployment of manipulative content in cognitive warfare.

#### 5.1.1 “Special operation” and nuclear energy

This cluster encapsulated narratives associating the Ukraine conflict with potential nuclear disasters. Aimed predominantly at European audiences, these stories sought to exploit fears related to energy security and nuclear safety, amplifying anxiety about the conflict's broader ramifications.

#### 5.1.2 “Kyiv authorities” and “dirty bomb”

Narratives in this cluster alleged that Ukraine was planning to deploy a “dirty bomb” to falsely implicate Russia in nuclear aggression. These claims aimed to delegitimize Ukraine while portraying Russia as a victim of unjust international accusations.

#### 5.1.3 Russia's potential nuclear strike

This cluster focused on narratives emphasizing Russia's nuclear capabilities and potential readiness to deploy nuclear weapons. These stories were strategically crafted to deter Western military interventions and project an image of Russian dominance and resolve.

#### 5.1.4 Nuclear programs and treaties

Discussions within this cluster revolved around international nuclear agreements, often highlighting alleged treaty violations or manipulations by Western powers. These narratives positioned Russia as an upholder of international norms, contrasting its actions with perceived breaches by adversaries.

#### 5.1.5 Navy fleet and blackmail

This cluster centered on narratives involving the Russian naval fleet and its potential role in nuclear coercion or geopolitical blackmail. These stories emphasized Russia's strategic leverage and its ability to project power through its naval assets.

**Figure 2:**
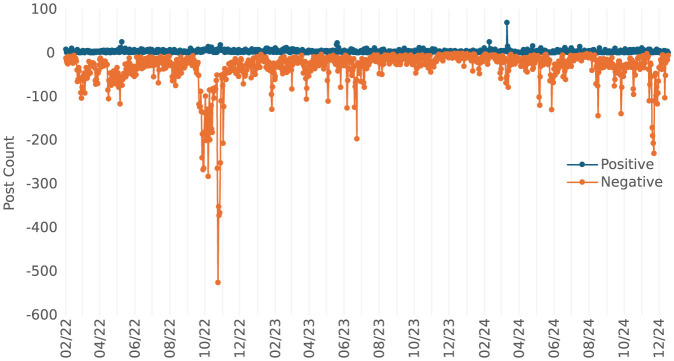
Sentiment analysis of the information dynamics of mentions of nuclear strikes.

Figure 2 illustrates the emotional tone of publications about nuclear threat narratives in the Russian information space during the analyzed period. This emotional profiling, derived using machine learning techniques, highlights the asymmetry in the emotional dynamics of the narratives, characterized by a predominance of negative (harmful) content.

- Negative publications: out of the 68,206 posts analyzed, 35,974 posts (52.7%) were classified as negative. This significant proportion underscores the deliberate use of fear, distrust, and anxiety to manipulate public sentiment.
- Positive publications: positive narratives accounted for only 3,506 posts (5.1%) of the content, reflecting minimal emphasis on optimistic or reassuring messaging within these narratives.

**Figure 3:**
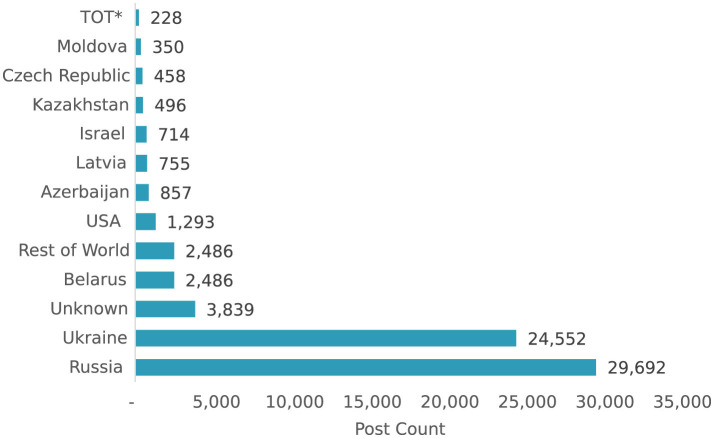
Post count by attributable country source.

Figure 3 shows the sources of analyzed posts by country where the country was determinable. TOT refers to Temporarily Occupied Territories of Ukraine. Unknown is used for analyzed posts where the country could not be accurately determined. Rest of World is a grouping of the remaining countries with post counts less than 350.

- Key narratives and themes: the attack-index tool revealed recurring motifs in Russian disinformation campaigns, such as nuclear threats, anti-NATO rhetoric, and victimization narratives. These narratives were often synchronized with geopolitical events to maximize impact. For instance:
- Nuclear threat narratives: Russian state-controlled media repeatedly emphasized the potential for nuclear conflict to deter Western support for Ukraine (NATO Allied Command Transformation, 2024). These narratives spiked during NATO summits and military aid announcements.
- Anti-NATO rhetoric: disinformation campaigns framed NATO as an aggressor, accusing the alliance of threatening Russian sovereignty (Brusylovska and Maksymenko, 2023; Benkő and Biczók, 2024).

Temporal analysis revealed that shifts in narrative tone coincided with key geopolitical events. For example, during high-profile NATO meetings, Russian narratives shifted from reassurance to hostility, correlating with announcements of increased military aid to Ukraine (Monaghan, 2020). Supplementing the above analysis, data extracted from the Attack Index database demonstrates a strong correlation between nuclear threat narratives and significant geopolitical meetings. In particular, the Attack Index values leading up to and following high-profile events such as the Ramstein meetings, NATO and EU summits, and G20 sessions suggest a shift in Russian disinformation tactics. The data reveals that the topic of nuclear strikes often gains resonance before these events, with the Attack Index values peaking just ahead of these critical moments. This pattern reflects an intentional effort to preemptively shape global discourse around nuclear escalation and influence international decision-making regarding military aid to Ukraine. Disinformation intensified during economic sanctions, leveraging themes of Western hypocrisy and Russian victimhood to bolster domestic support (NATO Allied Command Transformation, 2023; Deppe and Schaal, 2024). Russian state-controlled media persistently emphasizes nuclear threat narratives as a core component of its disinformation strategy. Analysis reveals that these narratives dominate both domestic and international segments of the Attack-Index database, reinforcing their importance in Russia's cognitive warfare arsenal between 2022 and 2024. This tactic underscores the Kremlin's reliance on nuclear rhetoric to manipulate perceptions and frame geopolitical discourse. The volume of nuclear threat mentions within Russian propaganda exhibits a resonant pattern, with significant peaks corresponding to pivotal geopolitical events. For example, during October 2022, mentions reached an absolute maximum of 807 instances per day, coinciding with heightened tensions in NATO-Ukraine relations. This temporal alignment indicates an intentional effort to exploit critical moments for maximum psychological impact.

**Figure 4:**
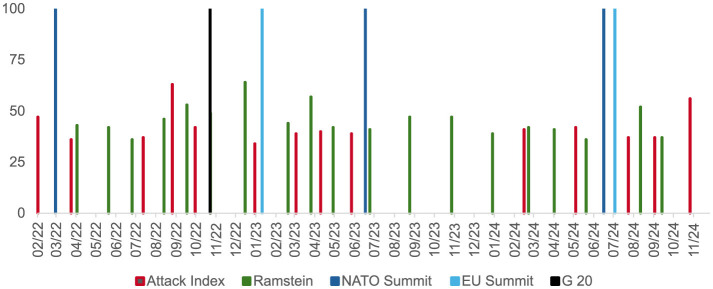
Temporal resonance values of the attack index over time, highlighting peaks in narrative activity preceding key geopolitical events.

Figure 4 illustrates the resonance values of the Attack Index on a temporal axis, plotted alongside information dynamics related to the Ramstein meetings, NATO and EU summits, and G20 sessions. The figure illustrates the strategic synchronization of disinformation campaigns with critical international milestones to maximize psychological and political impact. The data reveal a marked evolution in narrative synchronization. Note the blue and black columns that represent peaks in resonance values that occur predominantly before key international events, indicating a deliberate effort to shape narratives and public sentiment preemptively. This transition from reactive to proactive disinformation tactics underscores the adaptability of cognitive warfare strategies, particularly in leveraging nuclear rhetoric as a tool for psychological and strategic manipulation.

### 5.2 Correlation analysis and forecasting

The system conducted a correlation analysis to assess the relationship between mentions of nuclear threats and references to U.S. and NATO involvement in the Ukraine conflict. The analysis revealed that the highest correlation between these topics occurred in February 2021, with a Pearson correlation coefficient of 0.59. Forecast models projected increases in nuclear-related narratives, anticipating 600–700 daily mentions by March 2023. Following an escalation in nuclear threat rhetoric in October 2022, the correlation began to rise again, stabilizing at 0.45 by March 2023. This indicates a sustained link between discussions of nuclear threats and mentions of U.S. and NATO actions in the conflict. To complement this analysis, a forecasting model was developed using the Attack-Index system. The model projected a decline in nuclear threat mentions in early March 2023, followed by an increase, reaching 600–700 daily publications by the end of the month. This prediction utilized historical trends in mentions of nuclear threats alongside references to U.S. and NATO involvement.

### 5.3 AI-driven analysis

Purpose and Scope AI tools, including LLMs, were deployed to uncover nuanced manipulative tactics, such as sarcasm, euphemisms, and framing techniques. This approach enhanced the understanding of emotional and psychological appeals embedded in disinformation narratives. Cross-verification results from the Attack-Index tool were corroborated with AI-driven analyses to validate key insights. This integration provided a robust framework for understanding narrative construction and dissemination in cognitive warfare (Deppe and Schaal, 2024).

### 5.4 Key findings

Sentiment analysis of the nuclear threat narratives in Figure 2 highlights their predominantly negative tone. Approximately 52.7% of mentions expressed negative sentiment, compared to only 5.1% classified as positive. This deliberate focus on fear-based messaging exemplifies Russia's strategy of leveraging existential threats to undermine public confidence and provoke anxiety within target audiences. Linguistic Patterns: both tools highlighted the consistent use of emotionally charged language and logical fallacies, such as false equivalencies, to influence public sentiment (Huang et al., 2024; Grindrod, 2024). Psychological Exploitation: narratives exploited cognitive biases, including confirmation bias and availability heuristics, ensuring resonance with target audiences (Rabb et al., 2021; Monaghan, 2020). Narratives targeting fear and moral outrage effectively shape public opinion, leveraging psychological triggers to enhance resonance and spread (Vosoughi et al., 2018). Fear and Anger: Russian narratives leveraged existential threats and moral outrage to evoke fear and anger. For instance, nuclear threat narratives targeted Western audiences to discourage military interventions (Weissmann et al., 2021). Moral Outrage: emotional framing, such as claims of NATO's moral hypocrisy, was amplified before pivotal geopolitical moments, influencing public perception (Grindrod, 2024). Euphemisms and Sarcasm: AI tools detected subtle manipulative language that obscured aggression while projecting victimhood. For example, references to “peacekeeping” operations masked overt military actions (Muñoz and Nuño, 2024). Framing Techniques: the strategic use of framing presented Russia's actions as defensive while portraying NATO as a destabilizing force (Henschke, 2025). Russia reframes its aggression by portraying itself as a defender and victim, shifting blame to Western support for Ukraine. This narrative strategy inverts the roles of aggressor and victim to justify its actions and weaken global opposition (Turska, 2024). Longitudinal Trends: analysis revealed that narrative themes evolved, adapting to audience receptivity and geopolitical dynamics (Henschke, 2025; Van der Klaauw, 2023).

### 5.5 Geopolitical synchronization

Russian disinformation narratives display a high degree of synchronization with major geopolitical events, strategically amplifying their psychological impact. AI-driven analyses identified significant increases in victimhood narratives following Western sanctions, with Russia framed as unfairly targeted by the international community (Plaza et al., 2023). Temporal analysis using AI tools revealed that shifts in disinformation narratives were closely tied to real-world events. Claims of Western duplicity were prominently emphasized before key international conferences, leveraging public distrust to undermine alliances. Analysis revealed narrative spikes coinciding with NATO summits and major sanctions announcements, as detailed in the timeline of narrative resonance (Figure 4). These alignments illustrate the calculated synchronization of disinformation efforts with critical geopolitical events. By 2024, this synchronization had become increasingly proactive, with disinformation campaigns peaking in advance of these events to shape public opinion preemptively. This strategic alignment allowed adversaries to control the narrative and influence decision-making processes ahead of time. The study further demonstrated that disinformation narratives are deliberately synchronized with specific geopolitical developments to enhance their resonance and psychological effect. For instance, during NATO summits, anti-NATO rhetoric spiked, portraying Western actions as deliberate provocations against Russian sovereignty (Benkő and Biczók, 2024). These findings underscore the sophisticated tactics employed in cognitive warfare, highlighting the need for enhanced detection and countermeasures to mitigate their impact.

## 6 Discussion

### 6.1 Key contributions to literature

The findings of this study contribute significantly to the evolving body of research on cognitive warfare and disinformation, offering novel insights into the mechanisms and impacts of manipulative narratives in modern geopolitical conflicts. By identifying key themes such as nuclear threats, anti-NATO rhetoric, and victimization, the study highlights their centrality in Russian disinformation campaigns. These narratives were not randomly deployed but strategically aligned with significant geopolitical events to amplify their psychological impact and influence public perception (Monaghan, 2020).

### 6.2 Psychological triggers and cognitive biases

Emotional triggers—such as fear, anger, and moral outrage—were systematically leveraged to manipulate public sentiment and polarize target audiences. These emotions were not merely incidental; they were deliberately crafted to exploit cognitive biases like confirmation bias and availability heuristics, which inherently predispose individuals to accept information that aligns with pre-existing beliefs or recent memories (Plaza et al., 2023; Grindrod, 2024). By examining the strategic use of these biases, the study advances theoretical understandings of how psychological vulnerabilities can be weaponized in cognitive warfare. The findings expand the theoretical understanding of cognitive warfare by highlighting its psychological and technological dimensions, particularly the interplay between emotional triggers and narrative framing (Van der Klaauw, 2023).

### 6.3 Advanced manipulative strategies

The application of AI-driven tools illuminated nuanced manipulative tactics employed in disinformation campaigns. These included the use of euphemisms to obscure intent, sarcasm to undermine credibility, and framing techniques that redefined aggression as victimhood. These advanced rhetorical strategies underscore the sophistication of modern cognitive warfare, where language becomes a tool for psychological subversion (Huang et al., 2024; Muñoz and Nuño, 2024).

### 6.4 Temporal and strategic dynamics

The findings revealed a clear synchronization of narrative shifts with critical geopolitical developments, such as NATO summits and announcements of military aid. For example, during NATO meetings, narratives shifted from passive to hostile tones, leveraging the event's visibility to maximize strategic impact. This temporal alignment underscores the calculated nature of cognitive operations, aiming to influence public opinion and policymaking during key geopolitical milestones (Benkő and Biczók, 2024).

### 6.5 Methodological innovations

The methodological framework of this study employs a dual-layered approach, integrating semantic analysis with AI-driven techniques, exemplified by the Attack Index. This innovative combination enables a nuanced understanding of narrative construction and thematic evolution while providing real-time insights into the emotional resonance of manipulative narratives. By identifying psychological triggers, such as fear and moral outrage embedded within disinformation campaigns, the research advances theoretical perspectives and offers practical roadmaps for crafting targeted counter-narratives and preemptive strategies. Through the analysis of historical data and current narrative dynamics, the study demonstrates the predictive capabilities of advanced analytics in identifying and mitigating disinformation. This proactive approach underscores the critical importance of real-time monitoring systems and international collaboration in addressing the transnational challenges posed by cognitive warfare, ensuring resilience in an evolving threat landscape. The integration of semantic analysis tools like the Attack Index with AI-driven methodologies provides a robust framework for analyzing disinformation narratives. Semantic tools systematically identified recurring themes and emotional triggers, while AI models unveiled deeper layers of manipulation, such as logical fallacies and rhetorical devices. Cross-verification through triangulation ensured the robustness and reliability of findings, mitigating potential biases inherent in automated tools and enhancing the validity of results (Abu Arqoub, 2023; Sarwono, 2022). By bridging qualitative and quantitative methodologies, the study addresses a critical gap in cognitive warfare research. It demonstrates the effectiveness of combining human-centered analysis with AI technologies to capture both the psychological and technological dimensions of manipulative narratives. This integration advances academic understanding and provides actionable insights for countering disinformation and fortifying defenses against cognitive warfare (Deppe and Schaal, 2024; Henschke, 2025).

### 6.6 Further research

Further research is needed to analyze disinformation narratives' emotional and psychological impact, focusing on the role of specific emotional triggers like hope, despair, or indignation in shaping public perception (Hoyle et al., 2023; Rabb et al., 2021). Examining the evolution of cognitive warfare strategies across different conflicts and cultural contexts can provide insights into the adaptability of manipulative narratives and their long-term effects on societal trust (Brusylovska and Maksymenko, 2023). Combining perspectives from psychology, linguistics, political science, and computer science can enrich the understanding of cognitive warfare, fostering innovative methodologies for analyzing and countering its effects (Grindrod, 2024; Henschke, 2025). Collaboration between academia, governments, and international organizations is essential for developing standardized frameworks to address the complex challenges posed by cognitive warfare. Future research should focus on crafting actionable policies that enhance societal resilience and foster international cooperation (Marsili, 2023; NATO Allied Command Transformation, 2023).

## 7 Practical implications

The findings of this study underscore the sophistication and strategic nature of modern cognitive warfare, offering significant practical implications for policymakers, organizations, and governments seeking to counter disinformation effectively. Findings from network visualizations (Appendix Figure 5) and sentiment trends (Figure 2), provide a roadmap for developing real-time monitoring systems and counter-narrative strategies tailored to evolving disinformation trends. By integrating semantic and AI-driven analyses, the research provides actionable insights into the detection, prevention, and mitigation of manipulative narratives. The deployment of tools like the Attack-Index in real-time monitoring systems emerges as a cornerstone for addressing the complex challenges posed by cognitive warfare in contemporary geopolitical contexts (Huang et al., 2024; Weissmann et al., 2021).

### 7.1 Detection and prevention

Enhanced analytical tools, such as the Attack-Index and AI-based algorithms, play a crucial role in identifying emotional triggers, thematic clusters, and shifts in disinformation narratives. These tools allow policymakers and practitioners to detect manipulative strategies early, preempting their psychological and societal impacts. For example, by identifying spikes in nuclear threat narratives or anti-NATO rhetoric, these tools can provide early warnings of targeted disinformation campaigns designed to exploit geopolitical tensions (Huang et al., 2024). Real-time detection of emotional triggers, such as fear or moral outrage, also enables governments and organizations to tailor timely responses, mitigating the resonance and spread of harmful narratives (Plaza et al., 2023).

### 7.2 Strategic response

The study highlights how narrative synchronization with critical geopolitical events—such as NATO summits, military aid announcements, or sanctions—enhances the psychological impact of disinformation. By analyzing the alignment between these events and narrative shifts, policymakers can develop targeted countermeasures. Strategic responses, such as public awareness campaigns and transparent communication strategies, can disrupt the momentum of manipulative narratives before they reach their peak impact. For instance, preemptive public disclosures and fact-checking initiatives during key geopolitical milestones can diminish the credibility of disinformation and foster public resilience against cognitive manipulation (Nimmo and Flossman, 2024).

### 7.3 Real-time monitoring and decision support

The integration of tools like the Attack-Index into real-time monitoring systems provides an invaluable resource for decision-makers. These systems can deliver actionable intelligence by tracking disinformation patterns and emotional appeals across digital platforms, enabling a proactive approach to cognitive warfare. For example, governments and international organizations could deploy real-time monitoring dashboards to analyze the spread of narratives during high-stakes negotiations or elections, informing strategic decisions and communication policies (NATO Allied Command Transformation, 2024; Index Systems Ltd., 2023).

### 7.4 International collaboration

The transnational nature of cognitive warfare necessitates a coordinated response that transcends national borders. This study underscores the need for international collaboration in developing standardized frameworks and tools for countering disinformation. Collaborative efforts among governments, academic institutions, and technology companies can enhance the effectiveness of countermeasures, such as joint fact-checking initiatives, shared monitoring platforms, and collective policy responses. For example, NATO's efforts to integrate cognitive warfare countermeasures into its broader strategic framework demonstrate the potential for multinational cooperation in addressing this evolving threat (NATO Allied Command Transformation, 2023; Nimmo and Flossman, 2024).

### 7.5 Leveraging advanced technologies

Future research and practical applications should explore the potential of advanced technologies, such as Generative Adversarial Networks (GANs) and deepfake detection tools, in both propagating and mitigating manipulative narratives. The Appendix documents the application of clustering algorithms that lays a foundation for future research on scalable AI-driven methodologies to combat cognitive warfare and disinformation. These technologies can either enhance or counteract cognitive warfare, depending on their governance and application. Policymakers must prioritize the development of ethical, efficient and effective AI workflow tools such as Opus that can identify sophisticated disinformation tactics, such as deepfakes or synthetic media, ensuring they do not exacerbate the challenges of cognitive warfare (Fagnoni et al., 2024).

### 7.6 Building societal resilience

Beyond technological interventions, fostering societal resilience against cognitive warfare is critical. Media literacy programs and public awareness campaigns can empower individuals to recognize and resist manipulative narratives. These initiatives should target vulnerable populations, such as youth and digitally marginalized communities, who are often disproportionately affected by disinformation. By promoting critical thinking skills and digital literacy, governments and organizations can reduce the societal impact of cognitive warfare, enhancing overall resilience (Sarwono, 2022; Wallenius, 2023).

## 8 Limitations

Despite this study's comprehensive framework and robust methodologies, several limitations must be acknowledged to contextualize the findings and inform future research. While extensive, the datasets used in this study are not immune to biases inherent in the sources from which they were derived. For instance, Russian state-controlled and social media platforms may reflect selective or exaggerated narratives intended to manipulate perceptions, potentially skewing the analysis. Additionally, the reliance on publicly available data excludes covert or less-detectable disinformation campaigns, which could provide further insights into manipulative strategies. These limitations highlight the need for more diverse and representative datasets to comprehensively understand cognitive warfare. Although advanced AI tools like Large Language Models (LLMs) and the Attack-Index offer significant advantages in detecting and analyzing disinformation, they have flaws. AI systems are susceptible to biases introduced during training, which may influence the interpretation of narratives and emotional triggers. Moreover, these tools may struggle to capture complex cultural nuances or context-specific manipulations, limiting their applicability across diverse geopolitical environments. The inability of current AI models to fully account for sarcasm, irony, or deeply embedded cultural references may result in partial or incomplete analyses. Measuring the long-term effects of cognitive warfare on societal trust, political stability, or international relations presents a significant challenge. The psychological and societal impacts of disinformation often manifest over extended periods, making it difficult to establish causal links between specific narratives and broader outcomes. Additionally, the interplay of multiple factors—economic conditions, political developments, and media ecosystems—further complicates the measurement of disinformation's long-term effects. The rapidly evolving nature of cognitive warfare presents another limitation. While this study captures current strategies and tools, adversaries continuously develop more sophisticated methods, such as Generative Adversarial Networks (GANs) and more advanced AI-driven disinformation techniques. As a result, the findings may have limited applicability as the tactics of cognitive warfare evolve. Future studies must remain adaptable, employing iterative methodologies to account for emerging technologies and shifting geopolitical landscapes. Ethical considerations, such as ensuring data privacy and avoiding the misuse of findings, imposed certain restrictions on the scope of this research. While these measures were necessary to uphold ethical standards, they may have limited access to sensitive or proprietary data that could have enriched the analysis. Operationally, integrating multiple analytical tools required significant computational and logistical resources, which could constrain scalability in broader applications.

## 9 Conclusion

This study provides a detailed exploration of cognitive warfare, focusing on its mechanisms, impacts, and strategic dimensions within the context of the Russia-Ukraine conflict. Employing a robust methodological framework that integrates semantic analysis tools like the Attack-Index and AI-driven approaches, the research sheds light on constructing and disseminating manipulative narratives. The findings illustrate the sophistication of modern cognitive warfare, characterized by emotional exploitation, narrative synchronization, and the strategic use of technological tools. This research contributes to the broader understanding of cognitive warfare as a critical element of contemporary hybrid conflicts by offering actionable insights into countering disinformation. In summary, this research makes significant contributions to the understanding of cognitive warfare, particularly its application during the Russia-Ukraine conflict.

The paper's key contributions include:

1. Establishing nuclear rhetoric as a strategic tool within manipulative narratives, highlighting its synchronization with geopolitical events such as NATO summits and military aid meetings.
2. Introducing an innovative analytical framework that combines semantic analysis and AI-driven methodologies, enhancing the detection and prediction of disinformation narratives.
3. Providing actionable insights for crafting countermeasures, with a focus on the importance of real-time monitoring systems and international collaboration to address the transnational challenges posed by cognitive warfare.

By addressing these dimensions, the research bridges critical gaps in understanding cognitive warfare's mechanisms, impacts, and countermeasures, offering both theoretical and practical advancements for mitigating disinformation in contemporary conflicts.

## Data Availability

The datasets presented in this study can be found in online repositories. The names of the repository/repositories and accession number(s) can be found in the article/Supplementary material.
